# The Small Molecule Inhibitor QLT0267 Radiosensitizes Squamous Cell Carcinoma Cells of the Head and Neck

**DOI:** 10.1371/journal.pone.0006434

**Published:** 2009-07-30

**Authors:** Iris Eke, Franziska Leonhardt, Katja Storch, Stephanie Hehlgans, Nils Cordes

**Affiliations:** OncoRay – Center for Radiation Research in Oncology, Medical Faculty Carl Gustav Carus, Dresden University of Technology, Dresden, Germany; Mizoram University, India

## Abstract

**Background:**

The constant increase of cancer cell resistance to radio- and chemotherapy hampers improvement of patient survival and requires novel targeting approaches. Integrin-Linked Kinase (ILK) has been postulated as potent druggable cancer target. On the basis of our previous findings clearly showing that ILK transduces antisurvival signals in cells exposed to ionizing radiation, this study evaluated the impact of the small molecule inhibitor QLT0267, reported as putative ILK inhibitor, on the cellular radiation survival response of human head and neck squamous cell carcinoma cells (hHNSCC).

**Methodology/Principal Findings:**

Parental FaDu cells and FaDu cells stably transfected with a constitutively active ILK mutant (FaDu-IH) or empty vectors, UTSCC45 cells, *ILK* ^floxed/floxed(fl/fl)^ and *ILK* ^−/−^ mouse fibroblasts were used. Cells grew either two-dimensionally (2D) on or three-dimensionally (3D) in laminin-rich extracellular matrix. Cells were treated with QLT0267 alone or in combination with irradiation (X-rays, 0–6 Gy single dose). ILK knockdown was achieved by small interfering RNA transfection. ILK kinase activity, clonogenic survival, number of residual DNA double strand breaks (rDSB; γH2AX/53BP1 foci assay), cell cycle distribution, protein expression and phosphorylation (e.g. Akt, p44/42 mitogen-activated protein kinase (MAPK)) were measured. Data on ILK kinase activity and phosphorylation of Akt and p44/42 MAPK revealed a broad inhibitory spectrum of QLT0267 without specificity for ILK. QLT0267 significantly reduced basal cell survival and enhanced the radiosensitivity of FaDu and UTSCC45 cells in a time- and concentration-dependent manner. QLT0267 exerted differential, cell culture model-dependent effects with regard to radiogenic rDSB and accumulation of cells in the G2 cell cycle phase. Relative to corresponding controls, FaDu-IH and *ILK* ^fl/fl^ fibroblasts showed enhanced radiosensitivity, which failed to be antagonized by QLT0267. A knockdown of ILK revealed no change in clonogenic survival of the tested cell lines as compared to controls.

**Conclusions/Significance:**

Our data clearly show that the small molecule inhibitor QLT0267 has potent cytotoxic and radiosensitizing capability in hHNSCC cells. However, QLT0267 is not specific for ILK. Further *in vitro* and *in vivo* studies are necessary to clarify the potential of QLT0267 as a targeted therapeutic in the clinic.

## Introduction

The clinical administration of molecular targeted therapies as monotherapy or in combination with radio- or chemotherapy is constantly rising. Promising results have already been obtained in chronic myeloid leukemia and gastrointestinal stromal tumors using the BCR-ABL small molecule inhibitor Gleevec® [1] or in head and neck cancers using the inhibitory epidermal growth factor receptor antibody Erbitux® [2]. In addition to the large plethora of resistance-mediating influences such as gain-of-function mutations in proto-oncogenes, elevated levels of hypoxia, and multidrug resistance by p-glycoprotein overexpression [3]–[5], the importance of adhesion-mediated drug resistance and adhesion-mediated radioresistance has recently come to light [6]–[8].

As part of focal adhesion complexes, Integrin-Linked Kinase (ILK) was recently introduced as potent cancer target [9]. ILK is ubiquitously expressed, β1 and β3 integrin-bound protein with critical functions in cytoskeletal integrin-actin connection and focal adhesion formation [10]. ILK contains a C-terminal protein kinase catalytic domain, a Pleckstrin homology-like domain and a N-terminal domain, which consists of five Ankyrin repeats [11], [12]. Latest observations showed a participation of the Ankyrin 2–5 of ILK in the interaction of ILK with the LIM (Lin-1, Isl-1, Mec-3)1 domain of the LIM-only adapter protein PINCH1 (particularly interesting new cysteine-histidine-rich protein) [12]. Although biological and biochemical studies support the view that ILK is fundamental for integrin-actin linkage and integrin-growth factor receptor interactions [10], [13]–[16], the protein kinase ability of ILK is still controversial, which resulted in the classification of ILK as pseudokinase [17], [18].

The current notion of ILK as therapeutic target arose from both *in vitro* and *in vivo* studies. *In vitro*, cell survival and proliferation were shown to be regulated by ILK especially via the Phosphatidylinositol-3 Kinase/Akt and Mitogen-Activated Protein Kinase (MAPK) cascade [14], [19], [20]. The knockdown of ILK or a pharmacological ILK inhibition using QLT0267 or one of its derivatives indicated strong effects on cell survival, proliferation and adhesion [21]–[23]. Our own studies, however, clearly showed a critical antisurvival role of ILK in cells exposed to ionizing radiation [20], [24]–[27]. Investigations using human A549 lung cancer cells, FaDu hHNSCC cells, human HL60 leukemia cells or mouse fibroblasts provided evidence that cells either overexpressing a constitutively active kinase form of ILK or expressing a wildtype form of ILK are significantly more sensitive to irradiation than their normal or ILK knockout counterparts, respectively.

Histological studies in a variety of human tumors exhibited an overexpression of ILK. Among these tumor entities were carcinomas from the ovary, colon, thyroid, melanoma, prostate, stomach and lung [13], [28]–[35]. A recent broad study using tissue microarray technique questioned these results by showing similar ILK expression in tumor tissue and normal tissue [36]. Intriguingly, the same study also demonstrated a significant reduction of ILK expression with increasing tumor grade of human kidney cancers. Hence, this work suggested that ILK is tightly associated with differentiation but not with dedifferentiation and/or carcinogenesis.

Owing to the controversial findings about the genetic and pharmacological targeting of ILK, this study was performed to evaluate the cytotoxic and radiosensitizing effects of the putative pharmacological ILK inhibitor QLT0267 on clonogenic survival and signal transduction of different hHNSCC cell lines. To more precisely mimic a physiological growth environment, the treatment was accomplished either when cells adhered to laminin-rich extracellular matrix (lrECM) precoated dishes or when cells were imbedded into a three-dimensional (3D) lrECM. From the presented observations a broad but not ILK-specific inhibitory spectrum can be concluded. QLT0267 exerted cytotoxic and radiosensitizing effects in the tested cell lines in a concentration- and time-dependent manner.

## Results

### QLT0267 inhibits ILK kinase activity in a cell line- and culture model-dependent manner

**Figure 1:**
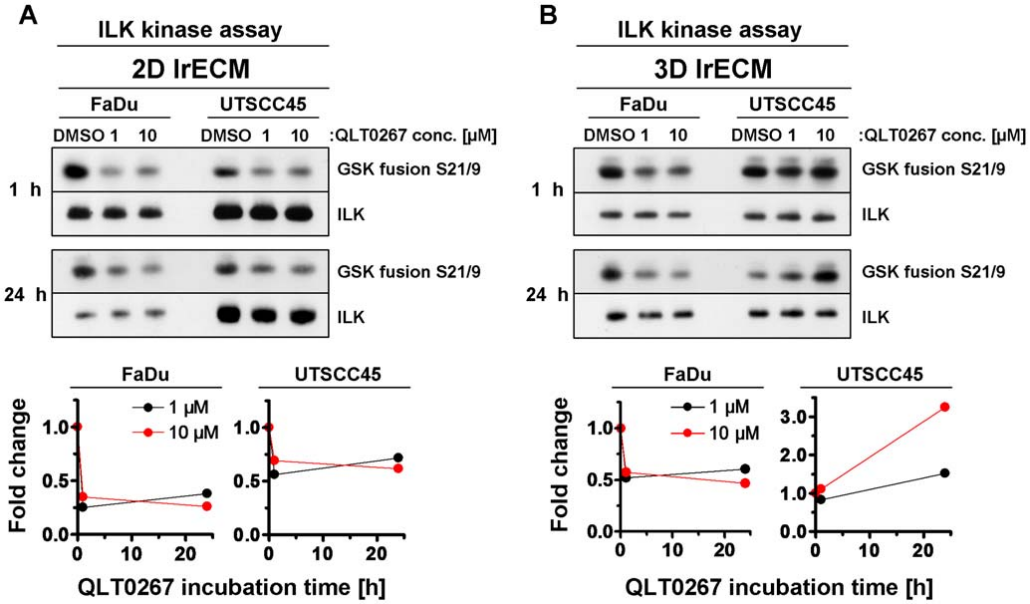
QLT0267 modifies ILK kinase activity in cell culture model-dependent manner. (A) 2D or (B) 3D cultured FaDu and UTSCC45 cells were exposed to 1 µM or 10 µM QLT0267 (DMSO served as control) and lysed after 1 h or 24 h. After immunoprecipitation of ILK and incubation with GSK fusion protein, samples were subjected to SDS-PAGE and Western blotting. Phosphorylation of GSK fusion protein with phospho-GSK3α/β S21/9 antibody indicates ILK kinase activity. Fold changes were calculated from densitometry and normalized to ILK and DMSO controls.

To assure efficient ILK kinase inhibition by QLT0267, we performed a kinase assay on immunoprecipitates from total cell lysates of 2D and 3D lrECM cell cultures treated with different QLT0267 concentrations over 1 h or 24 h. The data showed a strongly reduced ILK kinase activity in both 2D grown FaDu and UTSCC45 hHNSCC cell lines exposed to 1 µM QLT0267 as compared to an equivalent volume of DMSO (Figure 1A). No further ILK kinase inhibition was achieved when the dose was increased to 10 µM indicating that the maximal inhibitory effect of QLT0267 was already obtained at a concentration of 1 µM. In 3D, however, the inhibitory efficiency of QLT0267 on ILK was reduced in FaDu in comparison to 2D FaDu cell cultures, whereas in 3D UTSCC45 cells QLT0267 even exerted an inverse effect on ILK kinase activity relative to 2D conditions (Figure 1B).

### QLT0267 enhances the radiosensitivity of hHNSCC cells

**Figure 2:**
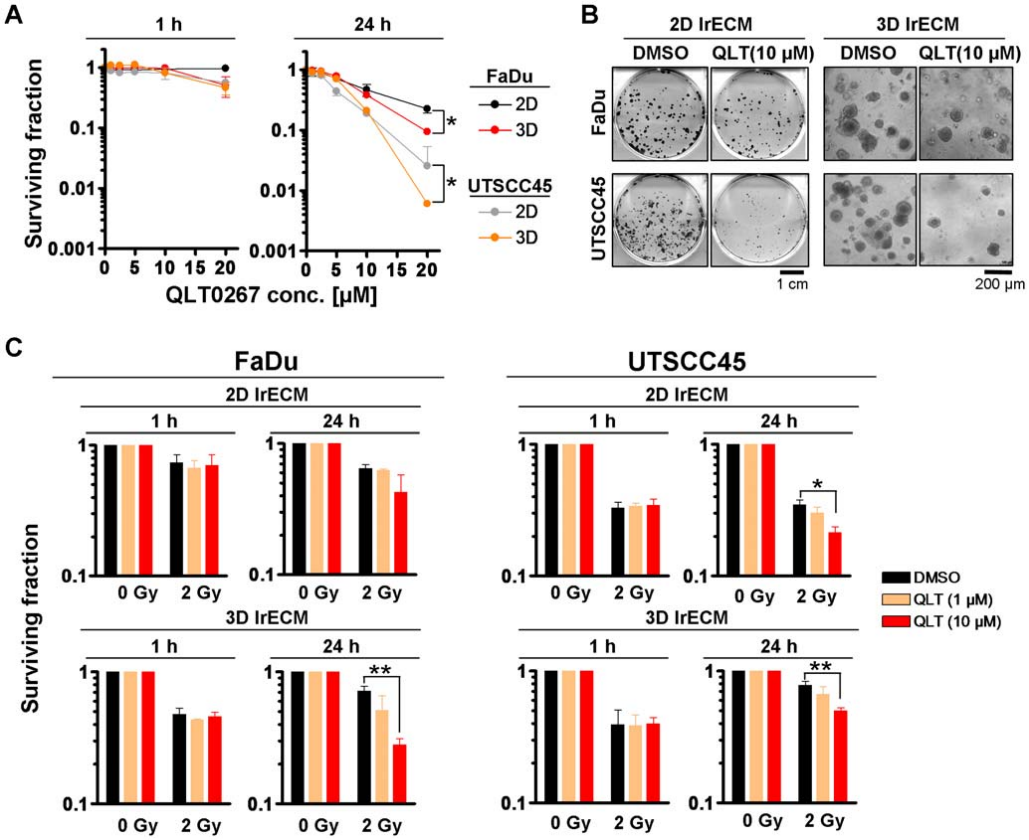
QLT0267 significantly reduces basal cell survival and sensitizes hHNSCC cells to ionizing radiation. (A) For 2D or 3D clonogenic assays, single cells were plated onto lrECM or inserted into lrECM and exposed to increasing concentrations of QLT0267 (0–20 µM) for 1 h or 24 h. Colonies were counted microscopically after 8–11 days. Results are means±s.d. (n = 3). Student's t-test compared clonogenic survival of QLT0267-treated cells under 2D vs. 3D growth conditions. *P<0.05. (B) Photographs show colonies 11 days (FaDu) or 8 days (UTSCC45) after treatment with DMSO or 10 µM QLT0267. (C) Subsequent to a 1-h or a 24-h exposure with DMSO or QLT0267, 2D and 3D cell cultures were irradiated with 2 Gy X-rays. Results represent means±s.d. (n = 3). Student's t-test compared QLT0267-treated/irradiated vs. DMSO-treated/irradiated cells. *P<0.05; **P<0.01

As QLT0267 modulated ILK kinase activity, we next analyzed the effect of QLT0267 on basal clonogenic survival and radiation clonogenic survival of FaDu and UTSCC45 hHNSCC cells under 2D and 3D cell culture conditions. On the basis of our previous studies [20], [25]–[27], we expected a radioprotective effect by QLT0267. A 1-h QLT0267 exposure showed no significant effect on both basal and radiation cell survival (Figure 2A-C, Table 1). Surprisingly, a 24-h QLT0267 treatment exerted significant (*P*<0.05) cytotoxicity (Fig. 2A and B) and radiosensitization of both FaDu and UTSCC45 cells to the clinical relevant radiation dose per fraction of 2 Gy relative to DMSO controls (Figure 2C, Table 1). Except in 3D FaDu cell cultures showing a 2.6-fold enhancement of the cellular radiosensitivity under QLT0267 exposure, 2D FaDu and 2D and 3D UTSCC45 cells were radiosensitized by QLT0267 by a factor of ∼1.5 (Table 1). Conclusively, neither the cytotoxic nor the radiosensitizing effects mediated by QLT0267 could be correlated with the changes in ILK kinase activity displayed in figure 1. Further, QLT0267 failed to show a radioprotective effect, which suggests a low or absent specificity for ILK.

**Table 1:** Calculation of enhancement ratios from survival rates of cells exposed to 2 Gy/DMSO versus 2 Gy/QLT0267 (10 µM).

	Enhancement Ratio SF (2 Gy)_DMSO_/SF(2 Gy)_QLT0267_
**FaDu 1h 2D**	1.04679
**FaDu 1h 3D**	1.03694
**FaDu 24h 2D**	1.512652
**FaDu 24h 3D**	2.570918
**UTSCC45 1h 2D**	0.954046
**UTSCC45 1h 3D**	0.978272
**UTSCC45 24h 2D**	1.617318
**UTSCC45 24h 3D**	1.574609

2D, two-dimensional cell culture; 3D, three-dimensional cell culture.

### Treatment with QLT0267 leads to an increase of radiation-induced residual DNA double strand breaks

**Figure 3:**
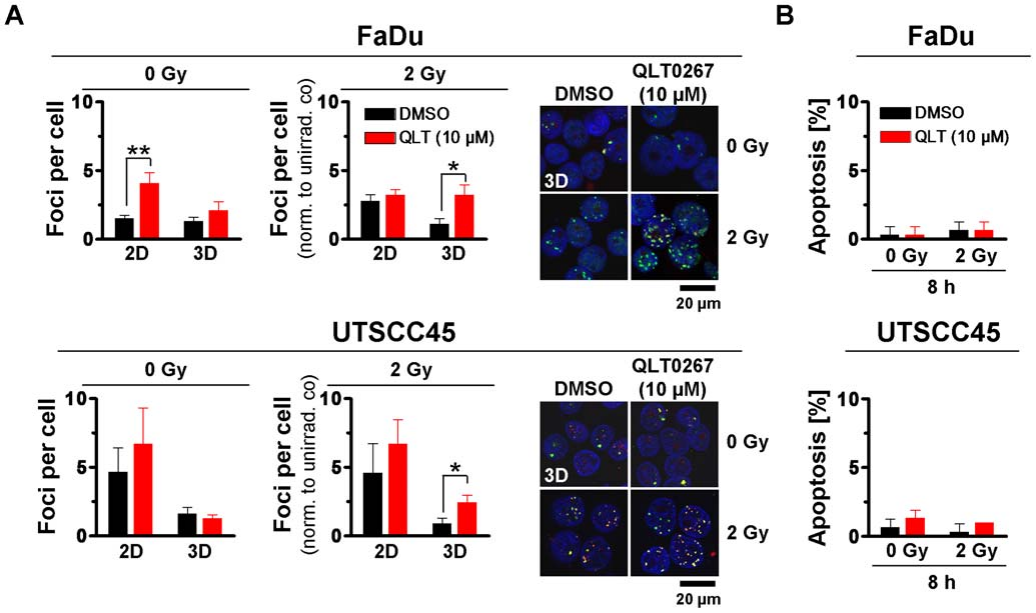
QLT0267 treatment increases the number of radiation-induced DSBs without affecting apoptosis. (A) After treatment with 10 µM QLT0267 for 24 h, 2D or 3D lrECM grown cells remained unirradiated or received a single dose of 2 Gy. After 24 h, cells were isolated, fixed and co-stained against 53BP1 and γH2AX. Double stained foci from 150 cells were microscopically counted per experiment. Number of foci of irradiated cells was normalized to number of foci of unirradiated cells. Results represent means±s.d. (n = 3). Student's t-test compared QLT0267- vs. DMSO-treated or QLT0267/2Gy- vs. DMSO/2Gy-treated cells. *P<0.05, **P<0.01. Photographs illustrate immunofluorescence staining of 53BP1 (green) and γH2AX (red) of 3D grown cell cultures. Nuclei were stained with DAPI (blue). (B) In parallel, cells were treated as indicated, fixed and stained with DAPI to microscopically determine cells with typically apoptotic nuclear morphology. Results are means±s.d. (n = 3).

DNA double strand breaks (DSB) are considered to be the most severe, life-threatening DNA lesions caused by ionizing radiation [37]. With regard to the cytotoxic and radiosensitizing effects of QLT0267, we measured the number of residual DSB ( = unrepaired DSB at 24 h after treatment; rDSB) induced by QLT0267 alone or in combination with 2-Gy irradiation using the γH2AX/53BP1 foci assay. Analysis of QLT0267 exposure only revealed a significant (*P*<0.01) elevation of rDSB in 2D grown FaDu cells (Figure 3A). In combination with irradiation, QLT0267-treated 3D FaDu and UTSCC45 cell cultures showed significantly (*P*<0.01) more rDSB 24 h after 2 Gy as compared to DMSO (Figure 3A).

### QLT0267 fails to induce apoptosis

Owing to reports showing induction of apoptosis by QLT0267, the rate of apoptosis was examined upon QLT0267 treatment with and without irradiation in comparison to DMSO controls. To note, p53 is deleted in FaDu and mutated in UTSCC45 cells (data not shown). QLT0267 alone and in combination with irradiation failed to induce apoptosis in 2D and 3D cultured FaDu and UTSCC45 cells (Figure 3B).

### QLT0267 treatment leads to accumulation of cells in the G2 cell cycle phase

**Figure 4:**
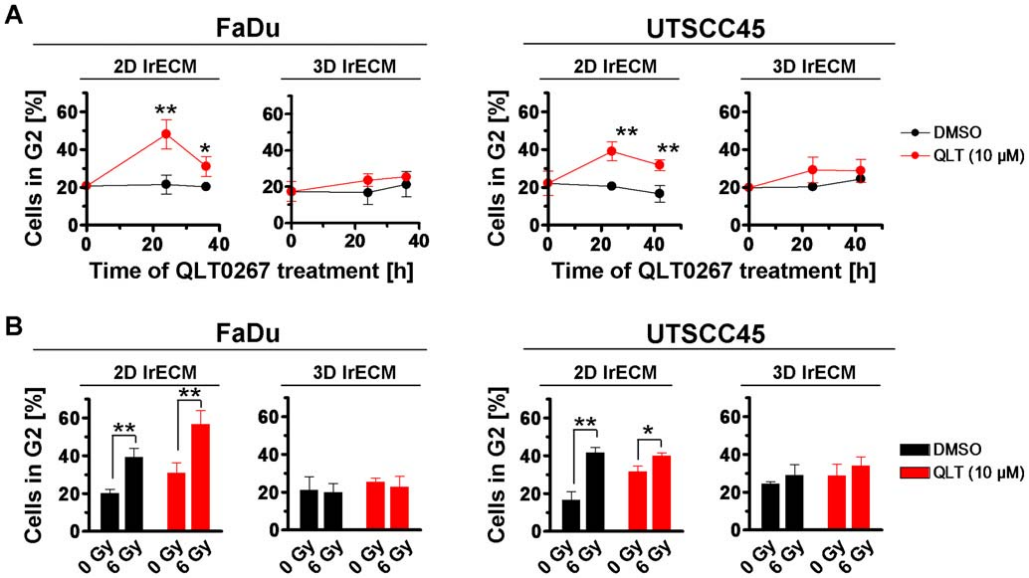
QLT0267 and irradiation induce accumulation of G2 phase cells in a cell culture model-dependent manner. (A) After treatment with QLT0267 for indicated time periods, cells were incubated with BrdU and cell cycle analysis was performed as described under Materials and Methods. Results are means±s.d. (n = 3). Student's t-test compared QLT0267- vs. DMSO-treated cells. *P<0.05; **P<0.01. (B) Cell cycle distribution was assayed in cells after a 24-h QLT0267 treatment plus 6 Gy X-rays (FaDu: 12 h post irradiation; UTSCC45: 18 h post irradiation). Results are means±s.d. (n = 3). Student's t-test compared QLT0267/irradiated vs. DMSO/irradiated cells. *P<0.05; **P<0.01.

As cell cycle phases can be associated with different degrees of radiosensitivity [37], [38], we next determined the percentage of cells in the radiosensitive G2 cell cycle phase upon QLT0267 alone or in combination with irradiation. The data indicated a significant (*P*<0.01) accumulation of G2 cells at 24 h after onset of QLT0267 treatment as compared to DMSO controls (Figure 4A). This effect could only be observed in 2D FaDu and 2D UTSCC45 cell cultures. Similarly, irradiation with 6 Gy resulted in a significant (*P*<0.01) G2 cell cycle blockage (Figure 4B). Intriguingly, the combination of QLT0267 plus 6 Gy showed a further significant (*P*<0.01) accumulation of cells in the G2 phase under 2D but not under 3D growth conditions (Figure 4B).

### Phosphorylation of Akt, GSK3β, FAK and p44/42 MAPK is modulated by QLT0267 in a cell culture model-dependent manner

**Figure 5:**
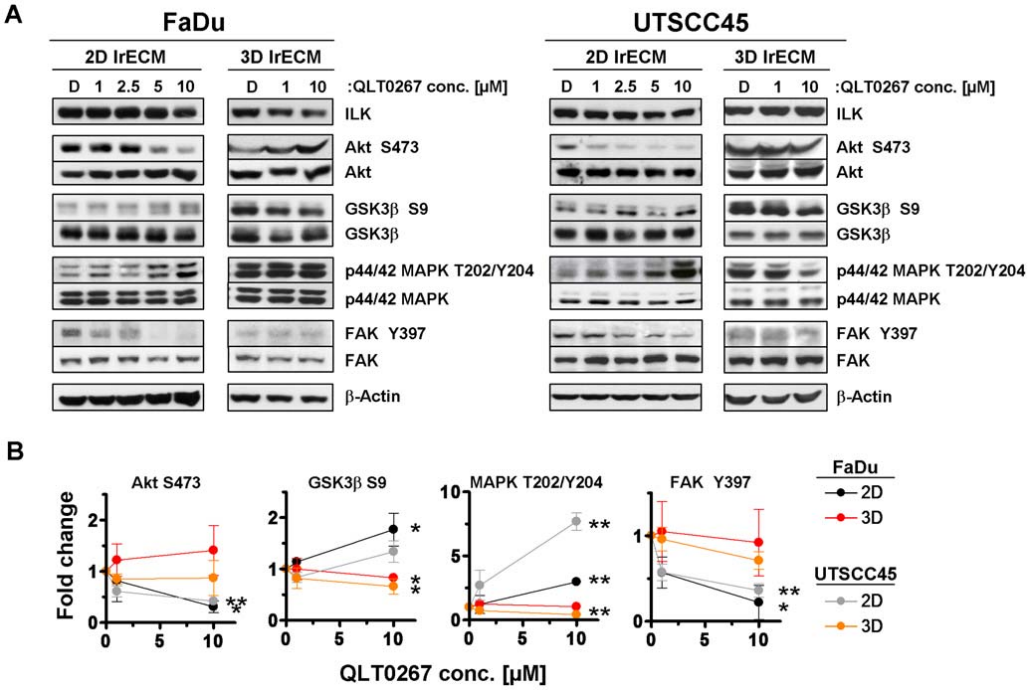
QLT0267 differentially modifies phosphorylation of various protein kinases cell culture model-dependently. (A) Following a 24-h treatment with DMSO (D) or indicated concentrations of QLT0267, 2D or 3D cultured cells were lysed as described under Materials and Methods. Total protein lysates were subjected to SDS-PAGE and Western blotting with specific antibodies. β-Actin served as loading control. (B) Protein phosphorylation was analyzed by densitometry and normalized to total protein expression. Results represent means±s.d. (n = 3). Student's t-test compared QLT0267- vs. DMSO-treated cells. *P<0.05; **P<0.01.

To examine the downstream effects of QLT0267 on important prosurvival signaling pathways, the phosphorylation and protein expression of ILK, FAK, p44/42 MAPK and the putative ILK downstream targets Akt and Glycogen Synthase Kinase 3β (GSK3β) were explored. Overall, the QLT0267-related modulation of examined protein kinases showed a great similarity between both cell lines (Figure 5A and B). In 2D, Akt Serine(S)473 and focal adhesion kinase (FAK) Tyrosine(Y)397 phosphorylation were strongly reduced by QLT0267 while both GSK3β S9 and p44/42 MAPK phosphorylation were moderately or greatly induced, respectively (Figure 5A and B). Although in part significant, the QLT0267-related changes in phosphorylation of examined protein kinases were lower under 3D conditions than under 2D conditions (Figure 5A and B). Expression of total proteins remained stable upon QLT0267 or DMSO treatment.

### Treatment with QLT0267 does not antagonize ILK-mediated radiosensitization

**Figure 6:**
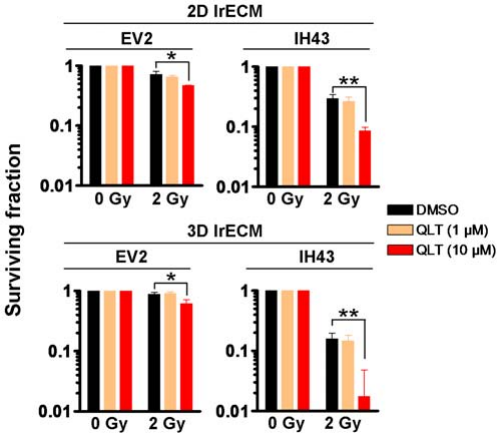
QLT0267 fails to antagonize the radiosensitization mediated by a constitutively active form of ILK. FaDu cells stably transfected with a constitutively active form of ILK (IH43) or control vector (EV2) were used. Cells cultured in 2D or 3D were exposed to 1 µM or 10 µM QLT0267 for 24 h and irradiated with 2 Gy X-rays. Results are means±s.d. (n = 3). Student's t-test compared QLT0267/2Gy- vs. DMSO/2Gy-treated cells. *P<0.05; **P<0.01.

Exogenous expression of a constitutively active form of ILK has been reported to enhance cellular radiosensitivity in a variety of human tumor cell models [20], [24], [26], [27]. Therefore, we sought to evaluate whether treatment of cells with the putative ILK inhibitor QLT0267 reverses ILK-mediated radiosensitization. Importantly, QLT0267 caused significant (*P*<0.01) additional cytotoxicity in FaDu cells expressing a constitutively active form of ILK (IH43 cells) and failed to diminish/antagonize the enhanced ILK-mediated radiosensitivity of these cells (Figure 6). From these findings we conclude that the radiosensitizing effects by QLT0267 are in majority independent from ILK.

### QLT0267 modulates radiation survival of mouse fibroblasts in an ILK-independent manner

**Figure 7:**
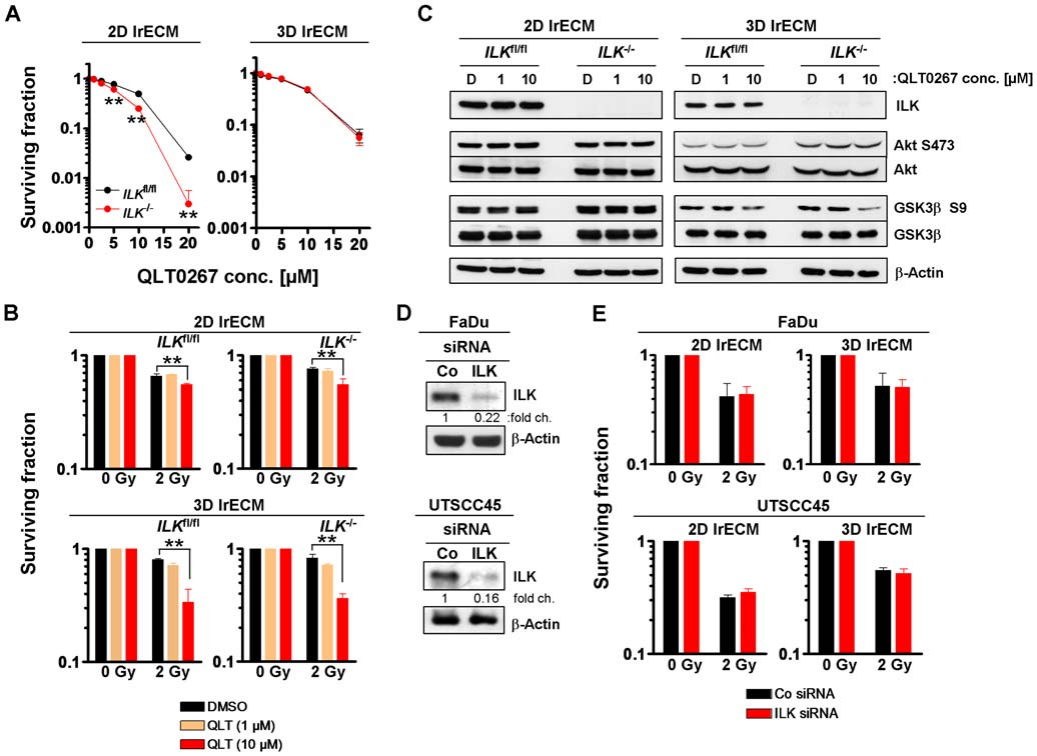
Modification of clonogenic survival and protein phosphorylation by QLT0267 are independent from ILK. Mouse *ILK* ^fl/fl^ and *ILK^−/−^* fibroblasts were cultured under 2D or 3D cell culture conditions, exposed to QLT0267 for 24 h plus/minus irradiation (2 Gy X-rays) and basal (A) and radiation cell survival (B) were measured as described under Materials and Methods. Results are means±s.d. (n = 3). Student's t-test compared QLT0267-treated *ILK* ^−/−^ vs. QLT0267-treated *ILK* ^fl/fl^ cells (A) or QLT0267/irradiated vs. DMSO/irradiated cells (B). *P<0.05; **P<0.01. (C) Total protein lysates from QLT0267- or DMSO-treated cells were analyzed by SDS-PAGE and Western blotting using indicated antibodies. (D) Cells were transfected with 20 nM ILK-specific siRNA or control siRNA. At 48 h post transfection, efficient downregulation of ILK expression was confirmed by Western blotting. Densitometric values were normalized to β-Actin. (E) For clonogenic assays, siRNA knockdown cell cultures were irradiated with 2 Gy X-rays 48 h after transfection. Results represent means±s.d. (n = 3).

To further clarify possible off-target effects of QLT0267, immortalized *ILK* ^−/−^ and ILK-expressing (*ILK* ^floxed/floxed(fl/fl)^) fibroblasts were exposed to QLT0267. Intriguingly, QLT0267 significantly (*P*<0.01) reduced basal cell survival in 2D and in 3D grown cells concentration-dependently (Figure 7A). *ILK* ^−/−^ fibroblasts demonstrated a higher susceptibility to QLT0267 than *ILK* ^fl/fl^ fibroblasts. The combination of QLT0267 with irradiation led to a significant (*P*<0.01) radiosensitization in 2D and 3D fibroblast cell cultures (Figure 7B). While the phosphorylation of the ILK putative downstream target Akt remained unaltered in 2D and 3D QLT0267-treated cultures, GSK3β S9 was marginally dephosphorylated under 3D growth conditions in *ILK* ^fl/fl^ and *ILK* ^−/−^ cells (Figure 7C). Total protein expression of ILK, Akt and GSK3β remained unaffected by QLT0267 and DMSO.

### siRNA-mediated knockdown of ILK does not alter the radiosensitivity of FaDu and UTSCC45 hHNSCC cells

Finally, we used a genetic, small interfering (si)RNA-based approach to eliminate ILK, which might exert different effects on radiosensitivity than a broad spectrum inhibitor such as QLT0267. The siRNA-mediated ILK knockdown (78–84% efficiency; Figure 7D) conveyed no significant changes in the radiosensitivity of 2D and 3D grown FaDu and UTSCC45 cells as compared to control siRNA (Figure 7E).

## Discussion

In combination with radiotherapy, ILK mediates rigorous antisurvival effects in a variety of human tumor cell models and in mouse fibroblasts [20], [24]–[27], [39]. Therefore, molecular targeting of ILK in patients receiving radiotherapy is highly questionable at the moment. *In vitro* and *in vivo* work from others [6], [10], [14], [16], [28], [40], [41], however, indicate a prosurvival and tumor-promoting function of ILK, which suggested ILK as potent molecular cancer target. Moreover, ILK has been considered as protein kinase, which fostered the development of small molecules against ILK such as QLT0254 [22], KP-SD-1 [40], KP-SD-2 [41] as well as the compound QLT0267 [23] used in this work. Preclinical studies with these components demonstrated tumor growth inhibition and induction of apoptosis of tumor cells. The present study explored, for the first time, the cytotoxic and radiosensitizing potential of the small molecule inhibitor QLT0267 and its dependence on ILK in hHNSCC cells and *ILK* ^fl/fl^ and *ILK* ^−/−^ mouse fibroblasts. To take growth conditions into account, we used conventional 2D/monolayer cell cultures grown on lrECM and more physiological 3D lrECM cell cultures [25], [42], [43]. The major results are that (i) QLT0267 effectively reduces ILK kinase activity as well as Akt and FAK phosphorylation in hHNSCC cells, (ii) QLT0267 enhances the radiosensitivity of hHNSCC cells and *ILK* ^fl/fl^ and *ILK* ^−/−^ mouse fibroblasts in an ILK-independent manner, (iii) QLT0267 increases the number of radiogenic residual DNA double strand breaks, (iv) QLT0267 fails to induce apoptosis in hHNSCC cells, (v) QLT0267 treatment leads to accumulation of 2D grown cells in the G2 cell cycle phase, and (vi) QLT0267 does not antagonize ILK-mediated radiosensitization.

In our hands, the small molecule inhibitor QLT0267 reduced ILK kinase activity efficiently by 50–75% in the two hHNSCC cell lines, FaDu and UTSCC45. Concerning the QLT0267-related 3-fold, unexpected increase in ILK activity in 3D UTSCC45 cell cultures, it can be speculated that such effects result from transcriptional and posttranscriptional modification of signaling pathways as well as from changes in protein-protein interactions induced by cell-matrix interactions in the 3D microenvironment. This adverse effect has also particularly to be taken into consideration on the basis of the inhibitory spectrum of QLT0267, which still needs to be determined. Without a correlation between ILK kinase inhibition and survival, QLT0267 treatment resulted in a concentration- and time-dependent reduction of clonogenic cell survival under both 2D and 3D growth conditions. In contrast to studies from other groups examining hHNSCC cell lines [23], a 24-h exposure with 10 µM QLT0267 did not lead to an elevated induction of apoptosis in FaDu and UTSCC45 cells, which could have been linked mechanistically to the declined clonogenic survival rate.

Intriguingly, the small molecule inhibitor QLT0267 sensitized the hHNSCC cell lines FaDu and UTSCC45 to the clinically relevant dose per fraction of 2 Gy X-rays. Although QLT0267-treated 2D cell cultures showed elevated numbers of γH2AX/p53BP1-positive foci, which might suggest a modulation of the DNA repair machinery, there appeared no correlation between foci number and radiosensitivity under 2D growth conditions. In contrast, foci number remained unchanged in 3D but significantly increased in irradiated 3D cell cultures suggesting an effect of QLT0267 on molecules involved in DNA repair or cell cycling. The cell cycle analysis revealed that merely 2D cell cultures exhibited a pronounced increase in G2 phase cells after QLT0267 incubation. Cells in the G2 cell cycle phase are considered more radiosensitive than cells e.g. in G1 [37], [38]. Thus, there appears no consistent, mechanistic link between the effects of QLT0267 on rDSB or cell cycling and radiosensitization. Nevertheless, our findings suggest QLT0267 as potent radiosensitizing agent with a yet to be determined inhibitory spectrum.

Our experiments shed light on this speculation by demonstrating that e.g. Akt dephosphorylation, as a published readout for ILK [14], followed a different pattern than the decrease in ILK kinase activity upon QLT0267. While ILK kinase activity is similarly reduced by 1 and 10 µM QLT0267, Akt dephosphorylation followed a concentration-effect relationship. The same holds true for FAK. Supporting data for this notion comes from our 3D cell cultures. Here, none of the investigated protein kinases showed a pronounced dephosphorylation. These findings, again, underscore the impact of a 3D growth environment on signaling cascades, on protein-protein interactions and on a modulated inhibitory efficacy of small molecules like QLT0267. Based on this, the molecular mechanisms whereby QLT0267 mediates its cytotoxic and radiosensitizing potential cannot be explained at the moment.

Further approaches exploring the effects of QLT0267 on radiosensitization employed FaDu cells expressing a constitutively active form of ILK and mouse *ILK* ^fl/fl^ and *ILK* ^−/−^ fibroblasts. If ILK mediates radiosensitization, a specific inhibition of ILK kinase activity through QLT0267 would result in radioprotection indicated by increased clonogenic cell survival. However, IH43 transfectants expressing a constitutively active form of ILK responded with enhanced radiosensitivity upon QLT0267. Accordingly, the comparison of *ILK* ^fl/fl^ and *ILK* ^−/−^ fibroblasts revealed a higher susceptibility of *ILK* ^−/−^ cells for QLT0267 in 2D but not in 3D. Interestingly, the QLT0267-mediated radiosensitization as well as the effects on phosphorylated S473 of Akt and S9 of GSK3β presented similar in *ILK* ^fl/fl^ and *ILK* ^−/−^ fibroblasts. Finally, clarification of the importance of ILK for the radiosensitivity of the tested hHNSCC cell lines in 2D and 3D was performed by ILK siRNA knockdown. Surprisingly, ILK depletion transduced no alterations of the clonogenic radiation survival in FaDu and UTSCC45 cells.

In conclusion, our data identified the small molecule inhibitor QLT0267 as potent therapeutic to enhance the cellular radiosensitivity of hHNSCC cells. Although strong evidence is presented that QLT0267 exerts these effects independent from ILK, further investigations are warranted to define the complete spectrum of this compound with regard to cancer target specificity and possible combinations with radiotherapy.

## Materials and Methods

### Antibodies and reagents

Antibodies against ILK, FAK, GSK3β, 5-bromo-2-deoxyuridine (BrdU) (BD, Heidelberg, Germany), phospho-Akt Serine(S)473, Akt, phospho-p44/42 MAPK, p44/42 MAPK, GSK3β S9, GSK3α/β S21/9 (Cell Signaling, Frankfurt a.M., Germany), phospho-FAK Tyrosine(Y)397 (Biosource, Solingen, Germany), β-Actin (Sigma, Taufkirchen, Germany), phospho-Histon H2AX-S139 (Upstate, Hamburg, Germany), p53 binding protein 1 (53BP1) (Novus, Littleton, USA), horseradish peroxidase-conjugated donkey anti-rabbit and sheep anti-mouse (Amersham, Freiburg, Germany), Alexa594 anti-mouse and Alexa488 anti-rabbit (Invitrogen, Karlsruhe, Germany) were purchased as indicated. Enhanced chemiluminescent reagent (ECL) was from Amersham, G418 from Calbiochem (Bad Soden, Germany), oligofectamine from Invitrogen and dimethyl sulfoxide (DMSO) from Applichem (Darmstadt, Germany). BrdU was from Serva (Heidelberg, Germany), RNase A type III-A from Sigma and pepsin 0.7 FIP-U from Merck (Darmstadt, Germany). Vectashield/DAPI mounting medium was from Alexis (Grünberg, Germany).

### Cell culture and radiation exposure

FaDu hHNSCC cells were kindly provided from M. Baumann (Dresden University of Technology, Germany) and stably transfected with pUSEamp vectors (Upstate, Hamburg, Germany) containing a cDNA construct encoding for ILK-hyperactive kinase (hk) or empty vector (pUSEamp) as published [27]. UTSCC45 hHNSCC cells were kindly provided from R. Grenman (Turku University Central Hospital, Finland). *ILK* ^fl/fl^ and *ILK* ^−/−^ immortalized mouse fibroblasts were kindly provided from R. Fässler (MPI, Martinsried, Germany; [44]). Cells were cultured in Dulbecco's Modified Eagle Medium (DMEM) containing glutamax-I supplemented with 10% fetal calf serum and 1% non-essential amino acids (PAA, Cölbe, Germany) at 37°C in a humidified atmosphere containing 10% CO_2_ (pH 7.4). In all experiments, asynchronously growing cells were used. Irradiation was delivered at room temperature using single doses of 200 kV X-rays (Yxlon Y.TU 320; Yxlon, Copenhagen, Denmark) filtered with 0.5 mm Cu. The absorbed dose was measured using a Duplex dosimeter (PTW, Freiburg, Germany). The dose-rate was approximately 1.3 Gy/min at 20 mA and applied doses ranged from 0 to 6 Gy.

### ILK kinase inhibition by QLT0267

QLT0267, obtained from QLT, Inc. (Vancouver, Canada), is an inhibitor of serine/threonine kinases with high preference for ILK [35]. QLT0267 was dissolved in DMSO to a concentration of 40 mM and stored at −80°C.

### Colony formation assay under 2D and 3D cell culture conditions

The 2D colony formation assay was applied for measurement of clonogenic cell survival as published [20]. Under two-dimensional conditions, cells were grown on laminin-rich extracellular matrix (lrECM, Matrigel; 1 µg/cm^2^; BD) for 24 h. Prior to irradiation with 0 to 2 Gy, cells were incubated with the small molecule QLT0267 (1–10 µM) or DMSO as control for 24 h. After 8 days, cells were stained with Coomassie blue and cell colonies (>50 cells) were counted. To evaluate clonogenic survival under three-dimensional conditions [25], single cells were grown in 0.5 µg/µl lrECM for 24 h prior to treatment with QLT0267 and irradiation. According to 2D clonogenic assays, cell clusters with a minimum of 50 cells were microscopically counted 8 to 11 days after plating. Plating efficiencies were calculated as follows: numbers of colonies formed/numbers of cells plated. Surviving fractions (SF) were calculated as follows: numbers of colonies formed/(numbers of cells plated (irradiated)×plating efficiency (unirradiated)). Each point on survival curves represents the mean surviving fraction from at least three independent experiments. Enhancement ratios were calculated as follows: SF (2 Gy)_DMSO_/SF(2 Gy)_QLT0267_.

### Total protein extracts and Western Blotting

Cells grown either on lrECM or in lrECM were incubated with QLT0267 (1–10 µM) or DMSO as control. In 2D, cells were rinsed with ice-cold PBS prior to adding modified RIPA buffer (50 mM Tris-HCl (pH 7.4), 1% Nonidet-P40, 0.25% sodium deoxycholate, 150 mM NaCl, 1 mM EDTA, Complete protease inhibitor cocktail (Roche, Mannheim, Germany), 1 mM NaVO_4_, 2 mM NaF) and samples were harvested by scraping. In 3D, cells were lysed in 3D lrECM using modified RIPA buffer. Samples were stored at −80°C. Total protein amounts were measured with the BCA assay (Pierce, Bonn, Germany). After SDS-PAGE and transfer of proteins onto nitrocellulose membranes (Schleicher and Schuell, Dassel, Germany), probing and detection of specific proteins was accomplished with indicated antibodies and ECL as described [24].

### ILK kinase activity assay

ILK kinase activity was determined as published previously [45]. In brief, cell lysis was performed using cell lysis buffer (Cell Signaling, Frankfurt a.M., Germany) supplemented with Complete protease inhibitor cocktail. Then, cells were scraped off and 500 µg of total protein were incubated with protein-G-agarose beads (Sigma, Taufkirchen, Germany) binding ILK antibody (BD, Heidelberg, Germany) for 24 h at 4°C. Immunoprecipitated ILK was used for protein kinase activity measurements using kinase buffer plus glycogen synthase kinase (GSK)-fusion protein and 200 µM adenosine triphosphate according to the manufacturer's protocol (Cell Signaling, Frankfurt a.M., Germany). After 30 min incubation at 30°C, the reaction was terminated with 3X sodium dodecyl sulfate (SDS) sample buffer. Subsequent to SDS-PAGE and Western blotting, ILK kinase activity was measured by detection of phosphorylated GSK fusion protein using GSK3α/β S21/9 antibodies.

### Immunofluorescence staining

For detection of residual DNA-double strand breaks (rDNA-DSBs) the phosphorylated H2AX (γH2AX)/p53 binding protein-1 (53BP1) focus assay [46] was performed. Cells were grown either on or in lrECM for 24 h, irradiated with 0 or 2 Gy and isolated 24 h thereafter using 5 mM EDTA/PBS and trypsin on ice. After cytospin, cells were fixed with 1% formaldehyde/PBS and permeabilized with 0.25% Triton X-100/PBS. Staining was accomplished with specific anti-γH2AX and anti-53BP1 antibodies and Vectashield/DAPI mounting medium. γH2AX/53BP1-positive nuclear foci of at least 150 cells from three independent experiments were counted microscopically with an Axioscope 2plus fluorescence microscope (Zeiss) and defined as rDSBs. Radiation-induced foci were calculated as follows: number of foci_irradiated_ – number of foci_unirradiated_.

### DAPI staining for apoptosis

Twenty-four hours prior to a 2-Gy radiation, cells were incubated with QLT0267 (10 µM) or DMSO. After 8 h, cells were washed with 0.9% NaCl (Roth, Karlsruhe, Germany), fixed and permeabilized using 4% paraformaldehyde/0.1% Triton X-100 (Merck, Darmstadt, Germany) and stained with Vectashield/DAPI mounting medium. At least 300 cells were counted from three independent experiments.

### siRNA-mediated ILK knockdown

ILK siRNA (sequence: 5′- GGGCAAUGACAUUGUCGUGtt -3′) was obtained from Applied Biosystems (Darmstadt, Germany). Ambion negative control siRNA #1 (Applied Biosystems) was used as nonspecific control. Cells were plated into 6-well plates 24 h prior to transfection. siRNA delivery was accomplished with oligofectamine under serum-free conditions at a concentration of 20 nM for 8 h as published [20]. Twenty-four hours after transfection, cells were trypsinized and transferred to lrECM either for colony formation assays or Western blotting.

### Cell cycle analysis

2D and 3D cell cultures were treated with 10 µM QLT0267 (or DMSO control), with 6 Gy X-rays or the combination of both. QLT0267-treated cells were harvested after a 24-h treatment time. Cells irradiated with 6 Gy were harvested after 12 h (FaDu) or 18 h (UTSCC45). In the combined regime, cells were harvested after 36 h (FaDu: 24-h QLT0267 plus 12 h 6 Gy) or 42 h (UTSCC45: 24-h QLT0267 plus 18 h 6 Gy). Prior to cell isolation with trypsin and 5 mM EDTA/PBS on ice, cells were incubated with 10 mM BrdU for 10 min (2D) or 30 min (3D). Then, cells were prepared for cell cycle analysis as published [24]. Detection of BrdU was accomplished with anti-BrdU and anti-mouse IgG FITC antibodies and total DNA staining with propidium iodide (PI) solution. Acquisition of data for 50,000 events was performed with a CyFlow (Partec, Münster, Germany). The distribution of cells in the different phases of the cell cycle was analyzed from the DNA-dot-blots and -histograms using FloMax software.

### Data analysis

Means±SD of at least three independent experiments were calculated with reference to untreated controls defined in a 1.0 scale. To test statistical significance, Student's *t* test was performed using Microsoft^®^ Excel 2003. Results were considered statistically significant if a *P*-value of less than 0.05 was reached.
